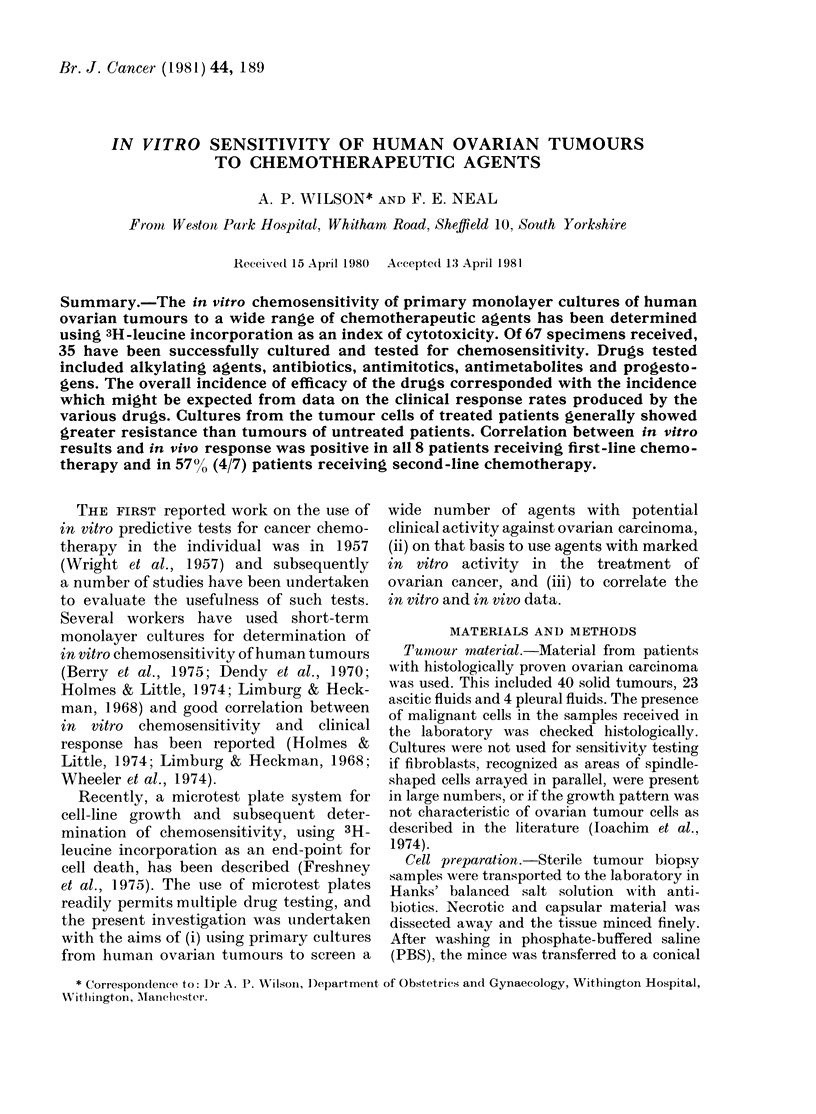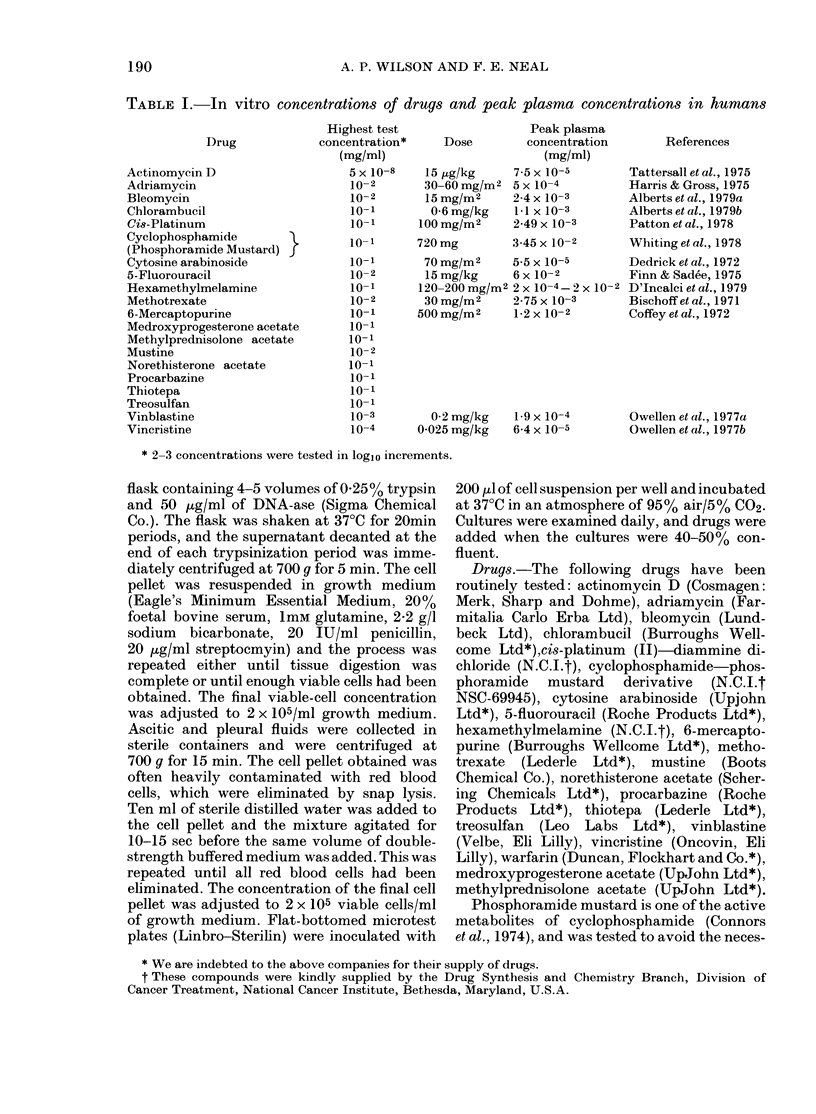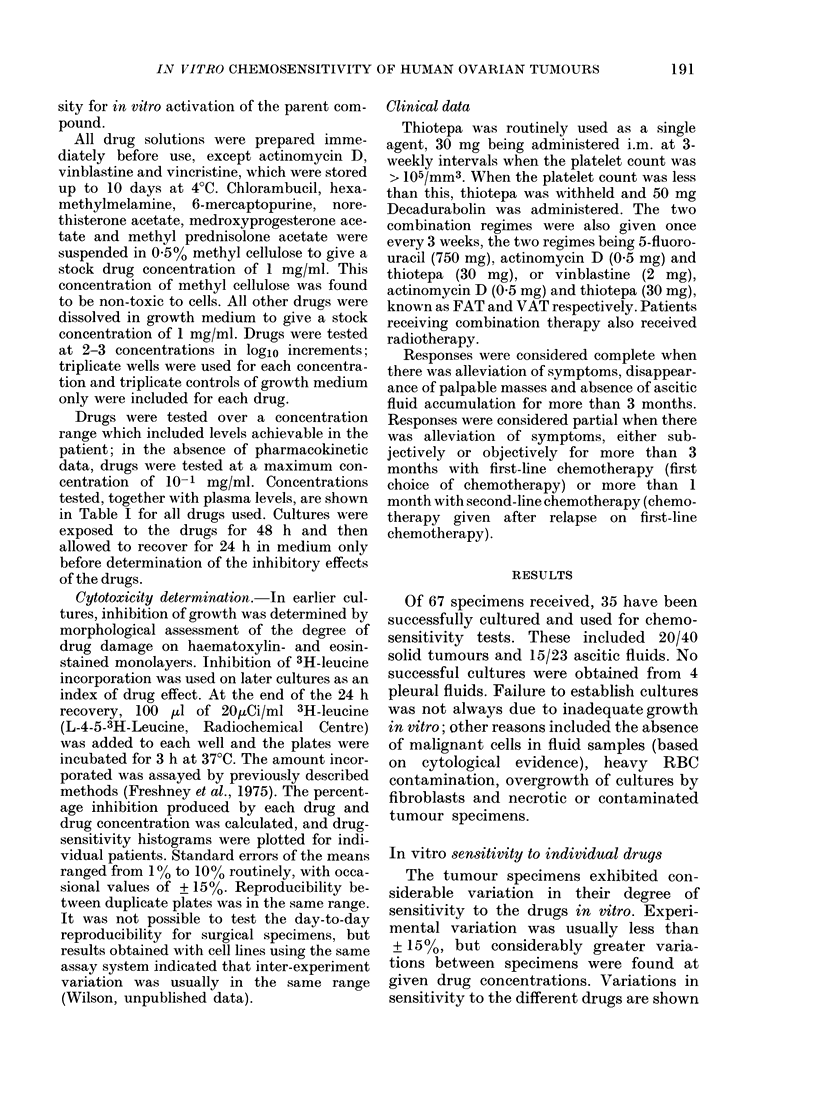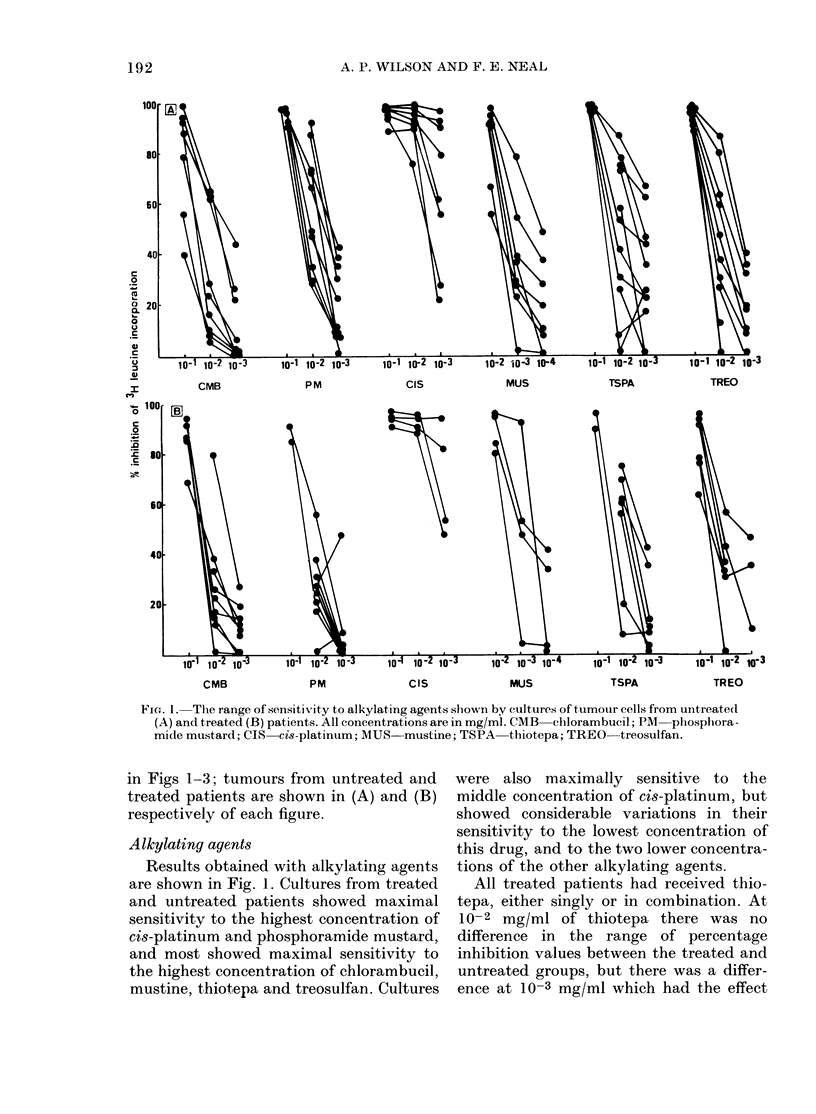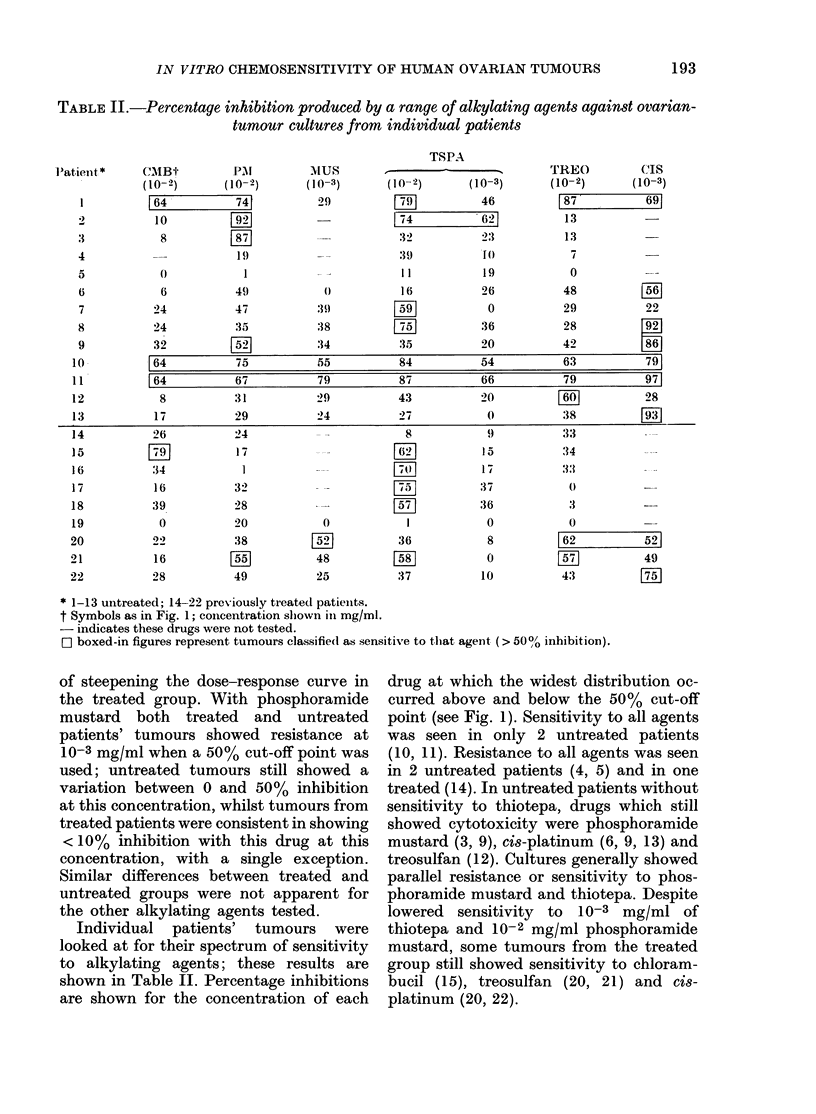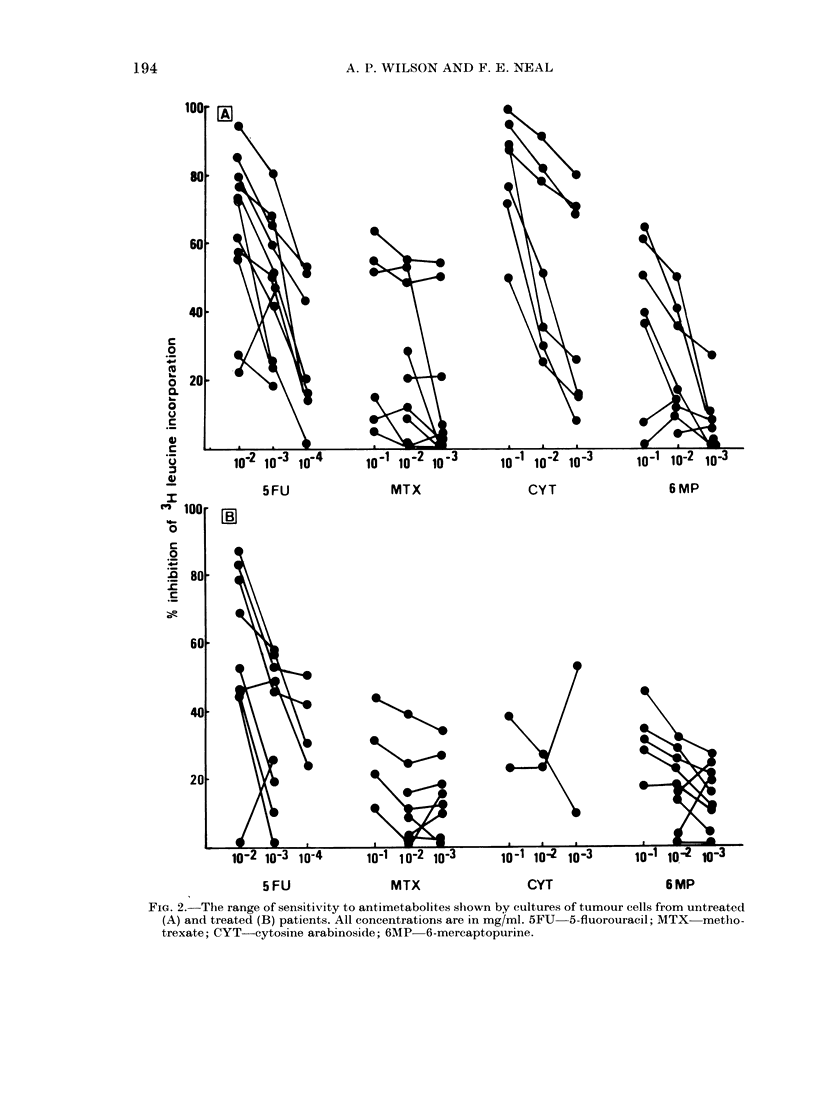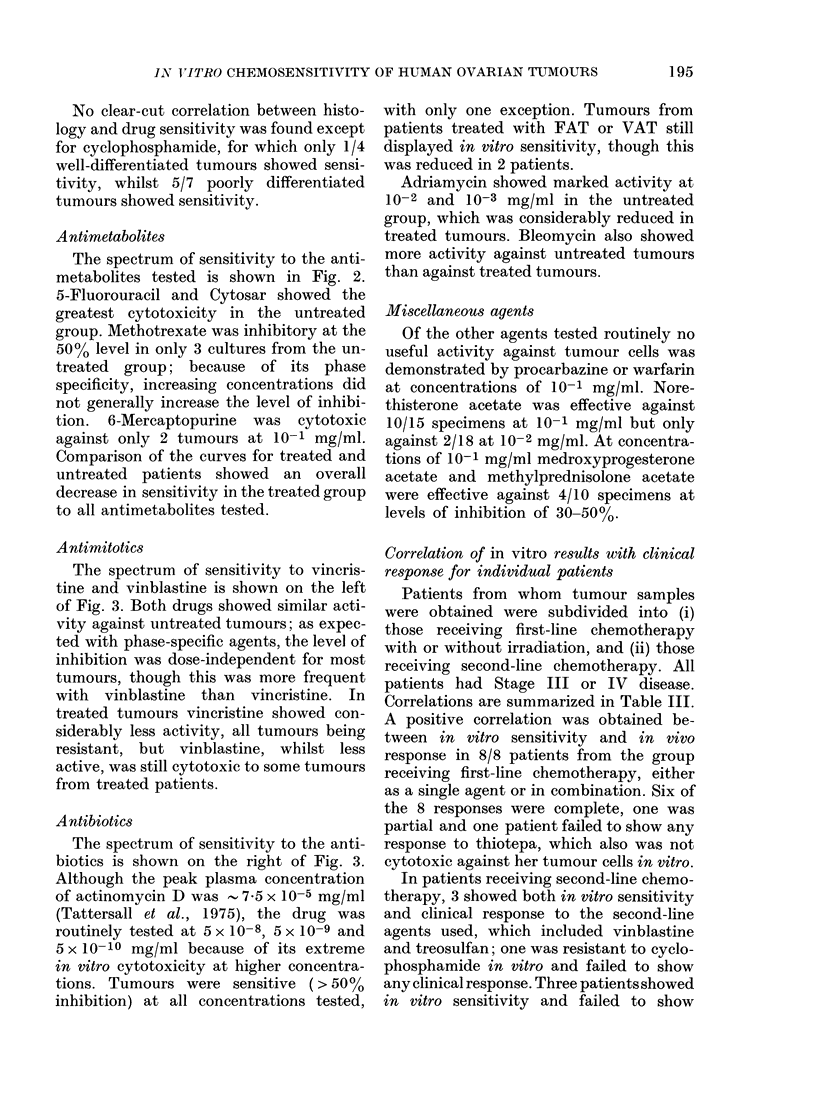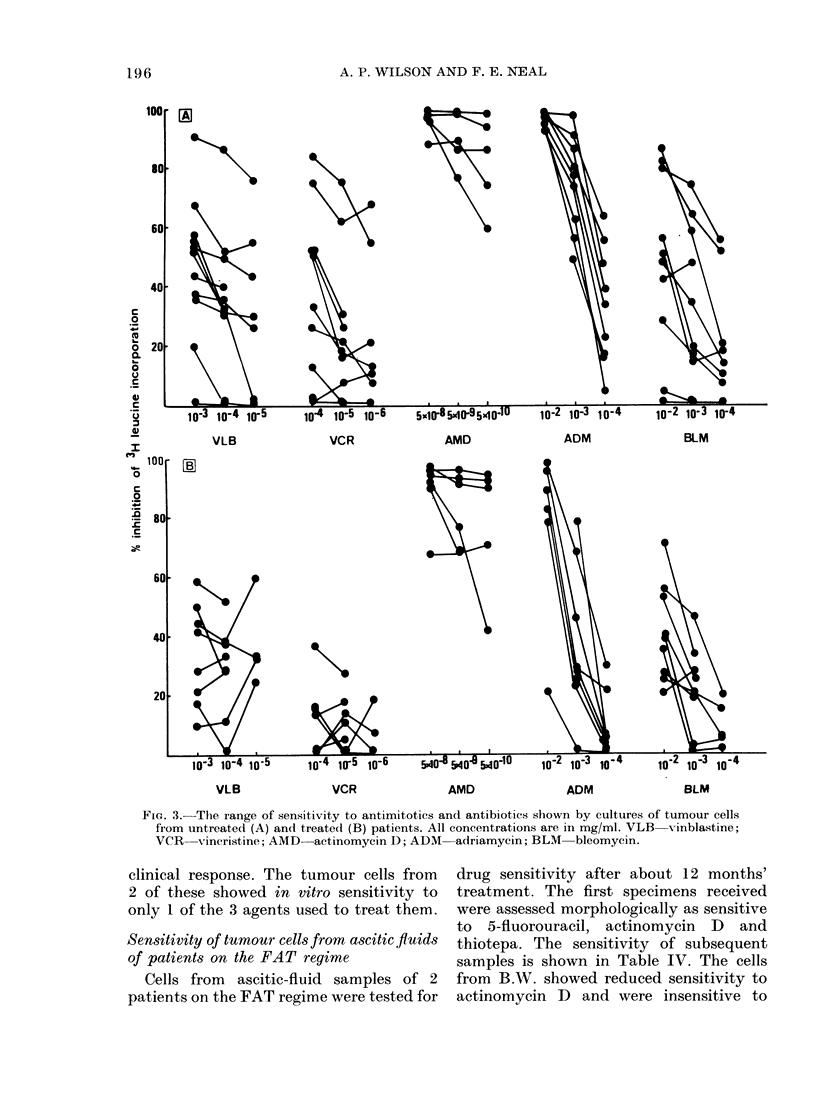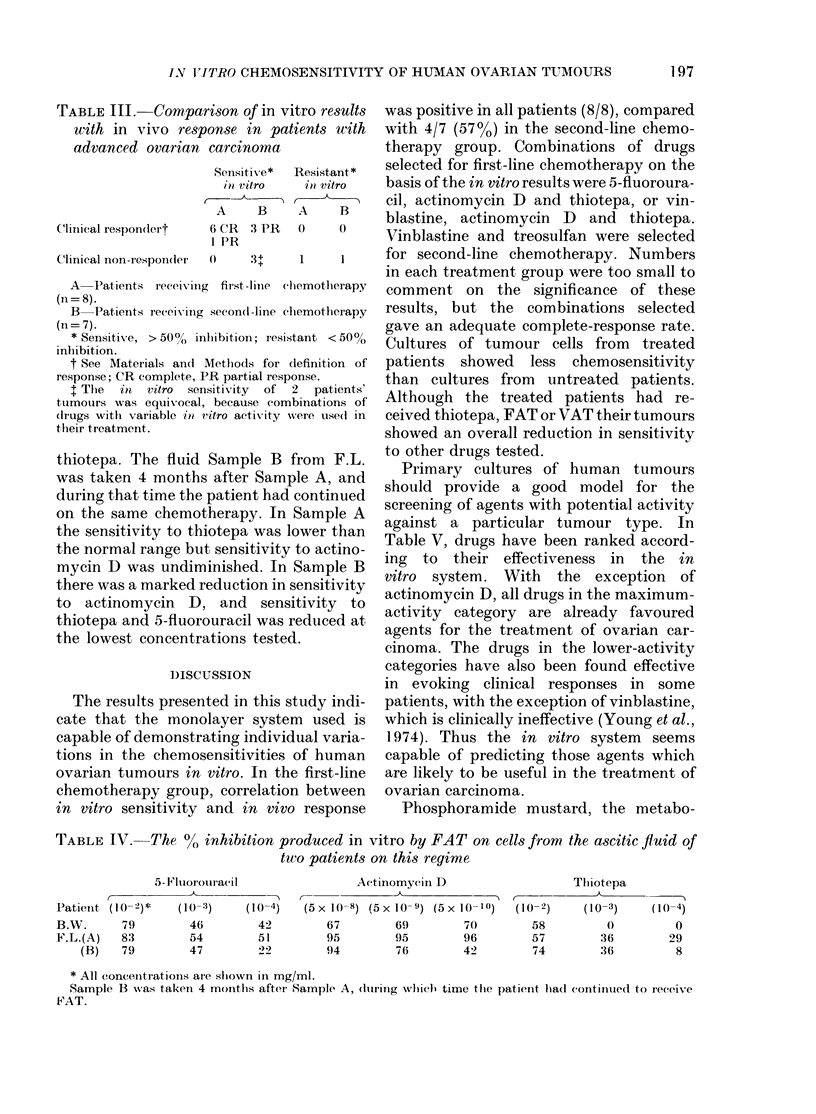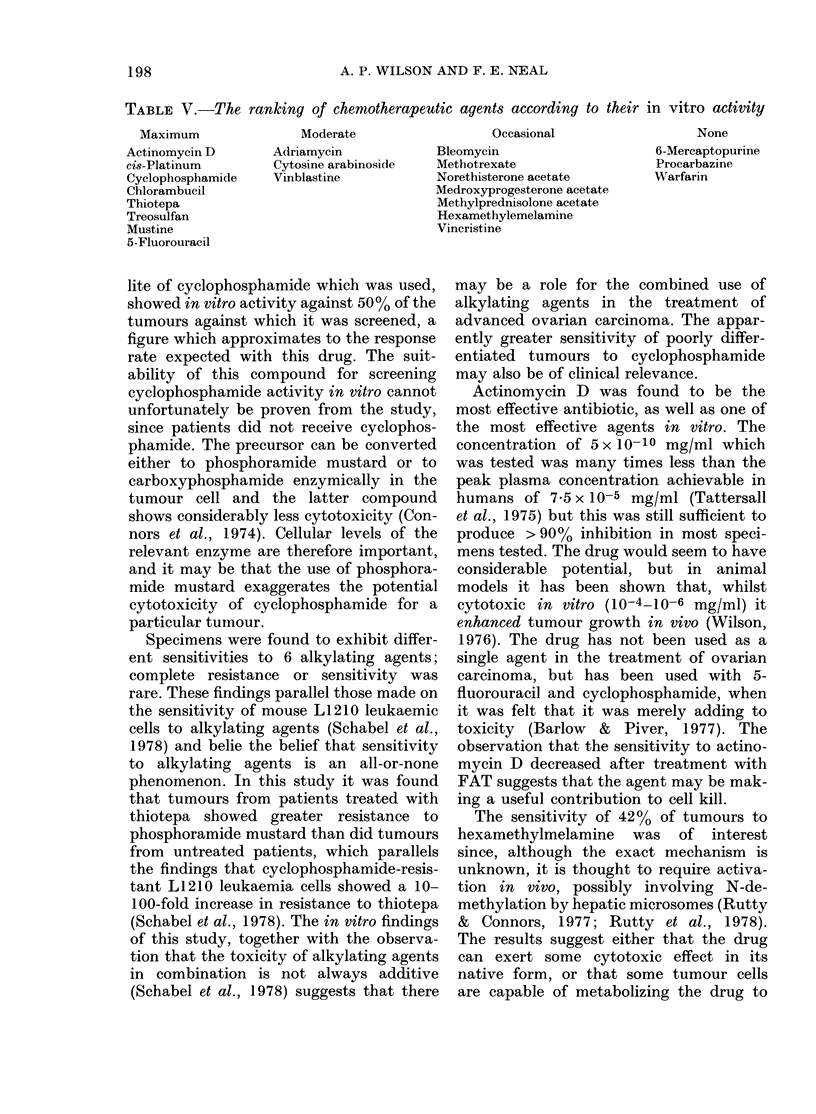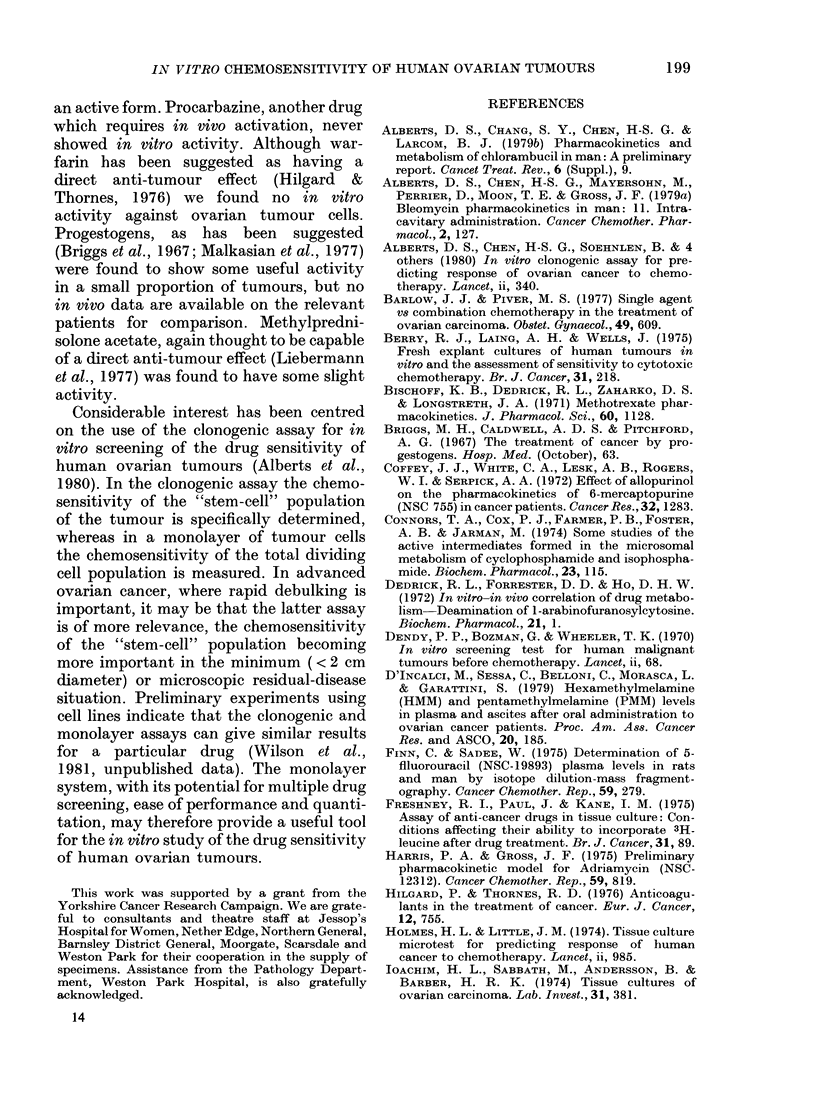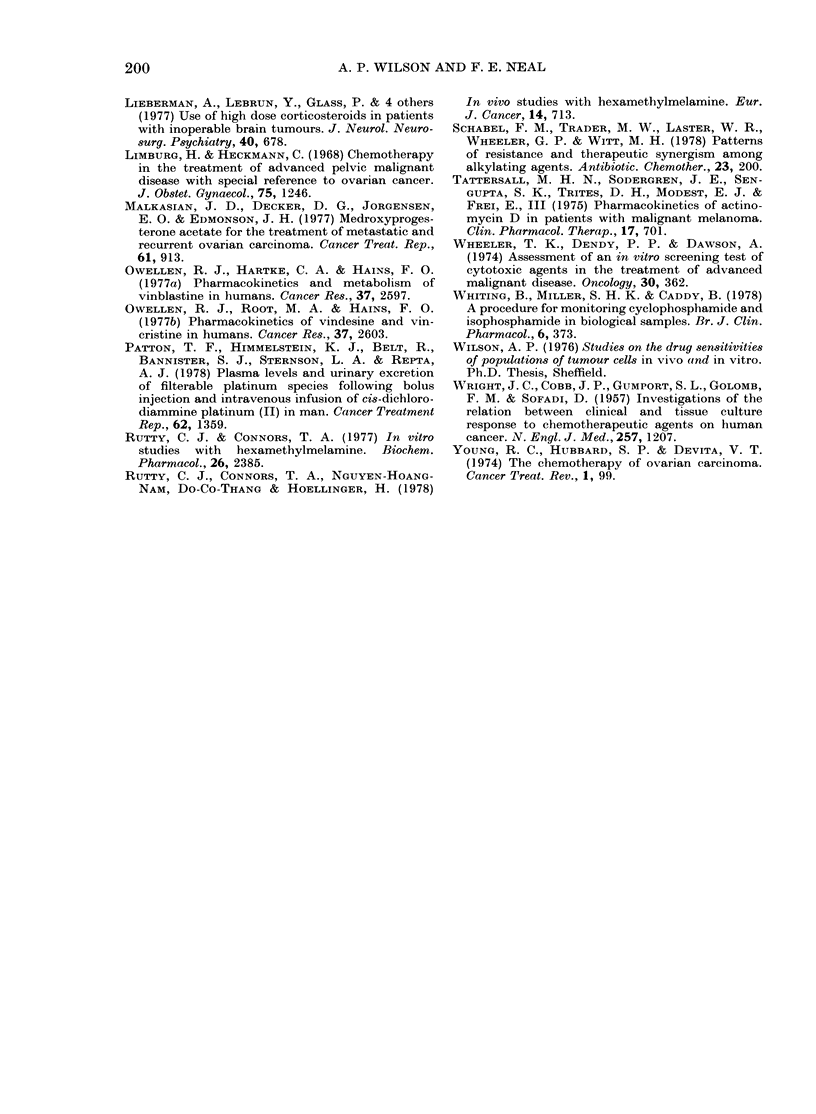# In vitro sensitivity of human ovarian tumours to chemotherapeutic agents.

**DOI:** 10.1038/bjc.1981.170

**Published:** 1981-08

**Authors:** A. P. Wilson, F. E. Neal

## Abstract

The in vitro chemosensitivity of primary monolayer cultures of human ovarian tumours to a wide range of chemotherapeutic agents has been determined using 3H-leucine incorporation as an index of cytotoxicity. Of 67 specimens received, 35 have been successfully cultured and tested for chemosensitivity. Drugs tested included alkylating agents, antibiotics, antimitotics, antimetabolites and progestogens. The overall incidence of efficacy of the drugs corresponded with the incidence which might be expected from data on the clinical response rates produced by the various drugs. Cultures from the tumour cells of treated patients generally showed greater resistance than tumours of untreated patients. Correlation between in vitro results and in vivo response was positive in all 8 patients receiving first-line chemotherapy and in 57% (4/7) patients receiving second-line chemotherapy.


*Br. J. Cancer* (1981) **44**, 189

### *IN VITRO* SENSITIVITY OF HUMAN OVARIAN TUMOURS TO CHEMOTHERAPEUTIC AGENTS

A. P. WILSON* AND F. E. NEAL

*From Weston Park Hospital, Whitham Road, Sheffield 10, South Yorkshire*

Received 15 April 1980 Accepted 13 April 1981

**Summary.—The *in vitro* chemosensitivity of primary monolayer cultures of human ovarian tumours to a wide range of chemotherapeutic agents has been determined using $^{3}H$-leucine incorporation as an index of cytotoxicity. Of 67 specimens received, 35 have been successfully cultured and tested for chemosensitivity. Drugs tested included alkylating agents, antibiotics, antimitotics, antimetabolites and progestogens. The overall incidence of efficacy of the drugs corresponded with the incidence which might be expected from data on the clinical response rates produced by the various drugs. Cultures from the tumour cells of treated patients generally showed greater resistance than tumours of untreated patients. Correlation between *in vitro* results and *in vivo* response was positive in all 8 patients receiving first-line chemotherapy and in 57% (4/7) patients receiving second-line chemotherapy.**

THE FIRST reported work on the use of *in vitro* predictive tests for cancer chemotherapy in the individual was in 1957 (Wright *et al.*, 1957) and subsequently a number of studies have been undertaken to evaluate the usefulness of such tests. Several workers have used short-term monolayer cultures for determination of *in vitro* chemosensitivity of human tumours (Berry *et al.*, 1975; Dendy *et al.*, 1970; Holmes & Little, 1974; Limburg & Heckman, 1968) and good correlation between *in vitro* chemosensitivity and clinical response has been reported (Holmes & Little, 1974; Limburg & Heckman, 1968; Wheeler *et al.*, 1974).

Recently, a microtest plate system for cell-line growth and subsequent determination of chemosensitivity, using $^{3}H$-leucine incorporation as an end-point for cell death, has been described (Freshney *et al.*, 1975). The use of microtest plates readily permits multiple drug testing, and the present investigation was undertaken with the aims of (i) using primary cultures from human ovarian tumours to screen a wide number of agents with potential clinical activity against ovarian carcinoma, (ii) on that basis to use agents with marked *in vitro* activity in the treatment of ovarian cancer, and (iii) to correlate the *in vitro* and *in vivo* data.

### MATERIALS AND METHODS

*Tumour material.*—Material from patients with histologically proven ovarian carcinoma was used. This included 40 solid tumours, 23 ascitic fluids and 4 pleural fluids. The presence of malignant cells in the samples received in the laboratory was checked histologically. Cultures were not used for sensitivity testing if fibroblasts, recognized as areas of spindle-shaped cells arrayed in parallel, were present in large numbers, or if the growth pattern was not characteristic of ovarian tumour cells as described in the literature (Ioachim *et al.*, 1974).

*Cell preparation.*—Sterile tumour biopsy samples were transported to the laboratory in Hanks' balanced salt solution with antibiotics. Necrotic and capsular material was dissected away and the tissue minced finely. After washing in phosphate-buffered saline (PBS), the mince was transferred to a conical

* Correspondence to: Dr A. P. Wilson, Department of Obstetrics and Gynaecology, Withington Hospital, Withington, Manchester.

TABLE I.—*In* vitro *concentrations of drugs and peak plasma concentrations in humans*

| Drug | Highest test concentration* (mg/ml) | Dose | Peak plasma concentration (mg/ml) | References |
|---|---|---|---|---|
| Actinomycin D | $5 \times 10^{-8}$ | 15 μg/kg | $7{\cdot}5 \times 10^{-5}$ | Tattersall *et al.*, 1975 |
| Adriamycin | $10^{-2}$ | 30–60 mg/m² | $5 \times 10^{-4}$ | Harris & Gross, 1975 |
| Bleomycin | $10^{-2}$ | 15 mg/m² | $2{\cdot}4 \times 10^{-3}$ | Alberts *et al.*, 1979*a* |
| Chlorambucil | $10^{-1}$ | 0·6 mg/kg | $1{\cdot}1 \times 10^{-3}$ | Alberts *et al.*, 1979*b* |
| *Cis*-Platinum | $10^{-1}$ | 100 mg/m² | $2{\cdot}49 \times 10^{-3}$ | Patton *et al.*, 1978 |
| Cyclophosphamide (Phosphoramide Mustard) | $10^{-1}$ | 720 mg | $3{\cdot}45 \times 10^{-2}$ | Whiting *et al.*, 1978 |
| Cytosine arabinoside | $10^{-1}$ | 70 mg/m² | $5{\cdot}5 \times 10^{-5}$ | Dedrick *et al.*, 1972 |
| 5-Fluorouracil | $10^{-2}$ | 15 mg/kg | $6 \times 10^{-2}$ | Finn & Sadée, 1975 |
| Hexamethylmelamine | $10^{-1}$ | 120–200 mg/m² | $2 \times 10^{-4} - 2 \times 10^{-2}$ | D'Incalci *et al.*, 1979 |
| Methotrexate | $10^{-2}$ | 30 mg/m² | $2{\cdot}75 \times 10^{-3}$ | Bischoff *et al.*, 1971 |
| 6-Mercaptopurine | $10^{-1}$ | 500 mg/m² | $1{\cdot}2 \times 10^{-2}$ | Coffey *et al.*, 1972 |
| Medroxyprogesterone acetate | $10^{-1}$ | | | |
| Methylprednisolone acetate | $10^{-1}$ | | | |
| Mustine | $10^{-2}$ | | | |
| Norethisterone acetate | $10^{-1}$ | | | |
| Procarbazine | $10^{-1}$ | | | |
| Thiotepa | $10^{-1}$ | | | |
| Treosulfan | $10^{-1}$ | | | |
| Vinblastine | $10^{-3}$ | 0·2 mg/kg | $1{\cdot}9 \times 10^{-4}$ | Owellen *et al.*, 1977*a* |
| Vincristine | $10^{-4}$ | 0·025 mg/kg | $6{\cdot}4 \times 10^{-5}$ | Owellen *et al.*, 1977*b* |

* 2–3 concentrations were tested in $\log_{10}$ increments.

flask containing 4–5 volumes of 0·25% trypsin and 50 μg/ml of DNA-ase (Sigma Chemical Co.). The flask was shaken at 37°C for 20min periods, and the supernatant decanted at the end of each trypsinization period was immediately centrifuged at 700 *g* for 5 min. The cell pellet was resuspended in growth medium (Eagle's Minimum Essential Medium, 20% foetal bovine serum, 1mM glutamine, 2·2 g/l sodium bicarbonate, 20 IU/ml penicillin, 20 μg/ml streptocmyin) and the process was repeated either until tissue digestion was complete or until enough viable cells had been obtained. The final viable-cell concentration was adjusted to $2 \times 10^5$/ml growth medium. Ascitic and pleural fluids were collected in sterile containers and were centrifuged at 700 *g* for 15 min. The cell pellet obtained was often heavily contaminated with red blood cells, which were eliminated by snap lysis. Ten ml of sterile distilled water was added to the cell pellet and the mixture agitated for 10–15 sec before the same volume of double-strength buffered medium was added. This was repeated until all red blood cells had been eliminated. The concentration of the final cell pellet was adjusted to $2 \times 10^5$ viable cells/ml of growth medium. Flat-bottomed microtest plates (Linbro–Sterilin) were inoculated with 200 μl of cell suspension per well and incubated at 37°C in an atmosphere of 95% air/5% $CO_2$. Cultures were examined daily, and drugs were added when the cultures were 40–50% confluent.

*Drugs*.—The following drugs have been routinely tested: actinomycin D (Cosmagen: Merk, Sharp and Dohme), adriamycin (Farmitalia Carlo Erba Ltd), bleomycin (Lundbeck Ltd), chlorambucil (Burroughs Wellcome Ltd*),*cis*-platinum (II)—diammine dichloride (N.C.I.†), cyclophosphamide—phosphoramide mustard derivative (N.C.I.† NSC-69945), cytosine arabinoside (Upjohn Ltd*), 5-fluorouracil (Roche Products Ltd*), hexamethylmelamine (N.C.I.†), 6-mercaptopurine (Burroughs Wellcome Ltd*), methotrexate (Lederle Ltd*), mustine (Boots Chemical Co.), norethisterone acetate (Schering Chemicals Ltd*), procarbazine (Roche Products Ltd*), thiotepa (Lederle Ltd*), treosulfan (Leo Labs Ltd*), vinblastine (Velbe, Eli Lilly), vincristine (Oncovin, Eli Lilly), warfarin (Duncan, Flockhart and Co.*), medroxyprogesterone acetate (UpJohn Ltd*), methylprednisolone acetate (UpJohn Ltd*).

Phosphoramide mustard is one of the active metabolites of cyclophosphamide (Connors *et al.*, 1974), and was tested to avoid the neces-

* We are indebted to the above companies for their supply of drugs.
† These compounds were kindly supplied by the Drug Synthesis and Chemistry Branch, Division of Cancer Treatment, National Cancer Institute, Bethesda, Maryland, U.S.A.

sity for *in vitro* activation of the parent compound.

All drug solutions were prepared immediately before use, except actinomycin D, vinblastine and vincristine, which were stored up to 10 days at 4°C. Chlorambucil, hexamethylmelamine, 6-mercaptopurine, norethisterone acetate, medroxyprogesterone acetate and methyl prednisolone acetate were suspended in 0·5% methyl cellulose to give a stock drug concentration of 1 mg/ml. This concentration of methyl cellulose was found to be non-toxic to cells. All other drugs were dissolved in growth medium to give a stock concentration of 1 mg/ml. Drugs were tested at 2–3 concentrations in $\log_{10}$ increments; triplicate wells were used for each concentration and triplicate controls of growth medium only were included for each drug.

Drugs were tested over a concentration range which included levels achievable in the patient; in the absence of pharmacokinetic data, drugs were tested at a maximum concentration of $10^{-1}$ mg/ml. Concentrations tested, together with plasma levels, are shown in Table I for all drugs used. Cultures were exposed to the drugs for 48 h and then allowed to recover for 24 h in medium only before determination of the inhibitory effects of the drugs.

*Cytotoxicity determination.*—In earlier cultures, inhibition of growth was determined by morphological assessment of the degree of drug damage on haematoxylin- and eosin-stained monolayers. Inhibition of $^3$H-leucine incorporation was used on later cultures as an index of drug effect. At the end of the 24 h recovery, 100 $\mu$l of 20$\mu$Ci/ml $^3$H-leucine (L-4-5-$^3$H-Leucine, Radiochemical Centre) was added to each well and the plates were incubated for 3 h at 37°C. The amount incorporated was assayed by previously described methods (Freshney *et al.*, 1975). The percentage inhibition produced by each drug and drug concentration was calculated, and drug-sensitivity histograms were plotted for individual patients. Standard errors of the means ranged from 1% to 10% routinely, with occasional values of $\pm 15\%$. Reproducibility between duplicate plates was in the same range. It was not possible to test the day-to-day reproducibility for surgical specimens, but results obtained with cell lines using the same assay system indicated that inter-experiment variation was usually in the same range (Wilson, unpublished data).

*Clinical data*

Thiotepa was routinely used as a single agent, 30 mg being administered i.m. at 3-weekly intervals when the platelet count was $> 10^5$/mm$^3$. When the platelet count was less than this, thiotepa was withheld and 50 mg Decadurabolin was administered. The two combination regimes were also given once every 3 weeks, the two regimes being 5-fluorouracil (750 mg), actinomycin D (0·5 mg) and thiotepa (30 mg), or vinblastine (2 mg), actinomycin D (0·5 mg) and thiotepa (30 mg), known as FAT and VAT respectively. Patients receiving combination therapy also received radiotherapy.

Responses were considered complete when there was alleviation of symptoms, disappearance of palpable masses and absence of ascitic fluid accumulation for more than 3 months. Responses were considered partial when there was alleviation of symptoms, either subjectively or objectively for more than 3 months with first-line chemotherapy (first choice of chemotherapy) or more than 1 month with second-line chemotherapy (chemotherapy given after relapse on first-line chemotherapy).

### RESULTS

Of 67 specimens received, 35 have been successfully cultured and used for chemosensitivity tests. These included 20/40 solid tumours and 15/23 ascitic fluids. No successful cultures were obtained from 4 pleural fluids. Failure to establish cultures was not always due to inadequate growth *in vitro*; other reasons included the absence of malignant cells in fluid samples (based on cytological evidence), heavy RBC contamination, overgrowth of cultures by fibroblasts and necrotic or contaminated tumour specimens.

### In vitro *sensitivity to individual drugs*

The tumour specimens exhibited considerable variation in their degree of sensitivity to the drugs *in vitro*. Experimental variation was usually less than $\pm 15\%$, but considerably greater variations between specimens were found at given drug concentrations. Variations in sensitivity to the different drugs are shown

FIG. 1.—The range of sensitivity to alkylating agents shown by cultures of tumour cells from untreated (A) and treated (B) patients. All concentrations are in mg/ml. CMB—chlorambucil; PM—phosphoramide mustard; CIS—*cis*-platinum; MUS—mustine; TSPA—thiotepa; TREO—treosulfan.

in Figs 1–3; tumours from untreated and treated patients are shown in (A) and (B) respectively of each figure.

*Alkylating agents*

Results obtained with alkylating agents are shown in Fig. 1. Cultures from treated and untreated patients showed maximal sensitivity to the highest concentration of *cis*-platinum and phosphoramide mustard, and most showed maximal sensitivity to the highest concentration of chlorambucil, mustine, thiotepa and treosulfan. Cultures were also maximally sensitive to the middle concentration of *cis*-platinum, but showed considerable variations in their sensitivity to the lowest concentration of this drug, and to the two lower concentrations of the other alkylating agents.

All treated patients had received thiotepa, either singly or in combination. At $10^{-2}$ mg/ml of thiotepa there was no difference in the range of percentage inhibition values between the treated and untreated groups, but there was a difference at $10^{-3}$ mg/ml which had the effect

TABLE II.—*Percentage inhibition produced by a range of alkylating agents against ovarian-tumour cultures from individual patients*

| Patient* | CMB† ($10^{-2}$) | PM ($10^{-2}$) | MUS ($10^{-3}$) | TSPA ($10^{-2}$) | TSPA ($10^{-3}$) | TREO ($10^{-2}$) | CIS ($10^{-3}$) |
|---|---|---|---|---|---|---|---|
| 1 | 64 | 74 | 29 | 79 | 46 | 87 | 69 |
| 2 | 10 | 92 | — | 74 | 62 | 13 | — |
| 3 | 8 | 87 | — | 32 | 23 | 13 | — |
| 4 | — | 19 | — | 39 | 10 | 7 | — |
| 5 | 0 | 1 | — | 11 | 19 | 0 | — |
| 6 | 6 | 49 | 0 | 16 | 26 | 48 | 56 |
| 7 | 24 | 47 | 39 | 59 | 0 | 29 | 22 |
| 8 | 24 | 35 | 38 | 75 | 36 | 28 | 92 |
| 9 | 32 | 52 | 34 | 35 | 20 | 42 | 86 |
| 10 | 64 | 75 | 55 | 84 | 54 | 63 | 79 |
| 11 | 64 | 67 | 79 | 87 | 66 | 79 | 97 |
| 12 | 8 | 31 | 29 | 43 | 20 | 60 | 28 |
| 13 | 17 | 29 | 24 | 27 | 0 | 38 | 93 |
| 14 | 26 | 24 | — | 8 | 9 | 33 | — |
| 15 | 79 | 17 | — | 62 | 15 | 34 | — |
| 16 | 34 | 1 | — | 70 | 17 | 33 | — |
| 17 | 16 | 32 | — | 75 | 37 | 0 | — |
| 18 | 39 | 28 | — | 57 | 36 | 3 | — |
| 19 | 0 | 20 | 0 | 1 | 0 | 0 | — |
| 20 | 22 | 38 | 52 | 36 | 8 | 62 | 52 |
| 21 | 16 | 55 | 48 | 58 | 0 | 57 | 49 |
| 22 | 28 | 49 | 25 | 37 | 10 | 43 | 75 |

* 1–13 untreated; 14–22 previously treated patients.
† Symbols as in Fig. 1; concentration shown in mg/ml.
— indicates these drugs were not tested.
□ boxed-in figures represent tumours classified as sensitive to that agent (>50% inhibition).

of steepening the dose–response curve in the treated group. With phosphoramide mustard both treated and untreated patients' tumours showed resistance at $10^{-3}$ mg/ml when a 50% cut-off point was used; untreated tumours still showed a variation between 0 and 50% inhibition at this concentration, whilst tumours from treated patients were consistent in showing <10% inhibition with this drug at this concentration, with a single exception. Similar differences between treated and untreated groups were not apparent for the other alkylating agents tested.

Individual patients' tumours were looked at for their spectrum of sensitivity to alkylating agents; these results are shown in Table II. Percentage inhibitions are shown for the concentration of each drug at which the widest distribution occurred above and below the 50% cut-off point (see Fig. 1). Sensitivity to all agents was seen in only 2 untreated patients (10, 11). Resistance to all agents was seen in 2 untreated patients (4, 5) and in one treated (14). In untreated patients without sensitivity to thiotepa, drugs which still showed cytotoxicity were phosphoramide mustard (3, 9), *cis*-platinum (6, 9, 13) and treosulfan (12). Cultures generally showed parallel resistance or sensitivity to phosphoramide mustard and thiotepa. Despite lowered sensitivity to $10^{-3}$ mg/ml of thiotepa and $10^{-2}$ mg/ml phosphoramide mustard, some tumours from the treated group still showed sensitivity to chlorambucil (15), treosulfan (20, 21) and *cis*-platinum (20, 22).

FIG. 2.—The range of sensitivity to antimetabolites shown by cultures of tumour cells from untreated (A) and treated (B) patients. All concentrations are in mg/ml. 5FU—5-fluorouracil; MTX—methotrexate; CYT—cytosine arabinoside; 6MP—6-mercaptopurine.

No clear-cut correlation between histology and drug sensitivity was found except for cyclophosphamide, for which only 1/4 well-differentiated tumours showed sensitivity, whilst 5/7 poorly differentiated tumours showed sensitivity.

*Antimetabolites*

The spectrum of sensitivity to the antimetabolites tested is shown in Fig. 2. 5-Fluorouracil and Cytosar showed the greatest cytotoxicity in the untreated group. Methotrexate was inhibitory at the 50% level in only 3 cultures from the untreated group; because of its phase specificity, increasing concentrations did not generally increase the level of inhibition. 6-Mercaptopurine was cytotoxic against only 2 tumours at $10^{-1}$ mg/ml. Comparison of the curves for treated and untreated patients showed an overall decrease in sensitivity in the treated group to all antimetabolites tested.

*Antimitotics*

The spectrum of sensitivity to vincristine and vinblastine is shown on the left of Fig. 3. Both drugs showed similar activity against untreated tumours; as expected with phase-specific agents, the level of inhibition was dose-independent for most tumours, though this was more frequent with vinblastine than vincristine. In treated tumours vincristine showed considerably less activity, all tumours being resistant, but vinblastine, whilst less active, was still cytotoxic to some tumours from treated patients.

*Antibiotics*

The spectrum of sensitivity to the antibiotics is shown on the right of Fig. 3. Although the peak plasma concentration of actinomycin D was $\sim 7{\cdot}5 \times 10^{-5}$ mg/ml (Tattersall *et al.*, 1975), the drug was routinely tested at $5 \times 10^{-8}$, $5 \times 10^{-9}$ and $5 \times 10^{-10}$ mg/ml because of its extreme *in vitro* cytotoxicity at higher concentrations. Tumours were sensitive ($>50\%$ inhibition) at all concentrations tested, with only one exception. Tumours from patients treated with FAT or VAT still displayed *in vitro* sensitivity, though this was reduced in 2 patients.

Adriamycin showed marked activity at $10^{-2}$ and $10^{-3}$ mg/ml in the untreated group, which was considerably reduced in treated tumours. Bleomycin also showed more activity against untreated tumours than against treated tumours.

*Miscellaneous agents*

Of the other agents tested routinely no useful activity against tumour cells was demonstrated by procarbazine or warfarin at concentrations of $10^{-1}$ mg/ml. Norethisterone acetate was effective against 10/15 specimens at $10^{-1}$ mg/ml but only against 2/18 at $10^{-2}$ mg/ml. At concentrations of $10^{-1}$ mg/ml medroxyprogesterone acetate and methylprednisolone acetate were effective against 4/10 specimens at levels of inhibition of 30–50%.

*Correlation of* in vitro *results with clinical response for individual patients*

Patients from whom tumour samples were obtained were subdivided into (i) those receiving first-line chemotherapy with or without irradiation, and (ii) those receiving second-line chemotherapy. All patients had Stage III or IV disease. Correlations are summarized in Table III. A positive correlation was obtained between *in vitro* sensitivity and *in vivo* response in 8/8 patients from the group receiving first-line chemotherapy, either as a single agent or in combination. Six of the 8 responses were complete, one was partial and one patient failed to show any response to thiotepa, which also was not cytotoxic against her tumour cells *in vitro*.

In patients receiving second-line chemotherapy, 3 showed both *in vitro* sensitivity and clinical response to the second-line agents used, which included vinblastine and treosulfan; one was resistant to cyclophosphamide *in vitro* and failed to show any clinical response. Three patients showed *in vitro* sensitivity and failed to show

FIG. 3.—The range of sensitivity to antimitotics and antibiotics shown by cultures of tumour cells from untreated (A) and treated (B) patients. All concentrations are in mg/ml. VLB—vinblastine; VCR—vincristine; AMD—actinomycin D; ADM—adriamycin; BLM—bleomycin.

clinical response. The tumour cells from 2 of these showed *in vitro* sensitivity to only 1 of the 3 agents used to treat them.

*Sensitivity of tumour cells from ascitic fluids of patients on the FAT regime*

Cells from ascitic-fluid samples of 2 patients on the FAT regime were tested for drug sensitivity after about 12 months' treatment. The first specimens received were assessed morphologically as sensitive to 5-fluorouracil, actinomycin D and thiotepa. The sensitivity of subsequent samples is shown in Table IV. The cells from B.W. showed reduced sensitivity to actinomycin D and were insensitive to

TABLE III.—*Comparison of* in vitro *results with* in vivo *response in patients with advanced ovarian carcinoma*

| | Sensitive* *in vitro* | | Resistant* *in vitro* | |
|---|---|---|---|---|
| | A | B | A | B |
| Clinical responder† | 6 CR 1 PR | 3 PR | 0 | 0 |
| Clinical non-responder | 0 | 3‡ | 1 | 1 |

A—Patients receiving first-line chemotherapy (n = 8).

B—Patients receiving second-line chemotherapy (n = 7).

* Sensitive, > 50% inhibition; resistant < 50% inhibition.

† See Materials and Methods for definition of response; CR complete, PR partial response.

‡ The *in vitro* sensitivity of 2 patients' tumours was equivocal, because combinations of drugs with variable *in vitro* activity were used in their treatment.

thiotepa. The fluid Sample B from F.L. was taken 4 months after Sample A, and during that time the patient had continued on the same chemotherapy. In Sample A the sensitivity to thiotepa was lower than the normal range but sensitivity to actinomycin D was undiminished. In Sample B there was a marked reduction in sensitivity to actinomycin D, and sensitivity to thiotepa and 5-fluorouracil was reduced at the lowest concentrations tested.

### DISCUSSION

The results presented in this study indicate that the monolayer system used is capable of demonstrating individual variations in the chemosensitivities of human ovarian tumours *in vitro*. In the first-line chemotherapy group, correlation between *in vitro* sensitivity and *in vivo* response was positive in all patients (8/8), compared with 4/7 (57%) in the second-line chemotherapy group. Combinations of drugs selected for first-line chemotherapy on the basis of the *in vitro* results were 5-fluorouracil, actinomycin D and thiotepa, or vinblastine, actinomycin D and thiotepa. Vinblastine and treosulfan were selected for second-line chemotherapy. Numbers in each treatment group were too small to comment on the significance of these results, but the combinations selected gave an adequate complete-response rate. Cultures of tumour cells from treated patients showed less chemosensitivity than cultures from untreated patients. Although the treated patients had received thiotepa, FAT or VAT their tumours showed an overall reduction in sensitivity to other drugs tested.

Primary cultures of human tumours should provide a good model for the screening of agents with potential activity against a particular tumour type. In Table V, drugs have been ranked according to their effectiveness in the *in vitro* system. With the exception of actinomycin D, all drugs in the maximum-activity category are already favoured agents for the treatment of ovarian carcinoma. The drugs in the lower-activity categories have also been found effective in evoking clinical responses in some patients, with the exception of vinblastine, which is clinically ineffective (Young *et al.*, 1974). Thus the *in vitro* system seems capable of predicting those agents which are likely to be useful in the treatment of ovarian carcinoma.

Phosphoramide mustard, the metabo-

TABLE IV.—*The % inhibition produced* in vitro *by FAT on cells from the ascitic fluid of two patients on this regime*

| | 5-Fluorouracil | | | Actinomycin D | | | Thiotepa | | |
|---|---|---|---|---|---|---|---|---|---|
| Patient | $(10^{-2})$* | $(10^{-3})$ | $(10^{-4})$ | $(5\times10^{-8})$ | $(5\times10^{-9})$ | $(5\times10^{-10})$ | $(10^{-2})$ | $(10^{-3})$ | $(10^{-4})$ |
| B.W. | 79 | 46 | 42 | 67 | 69 | 70 | 58 | 0 | 0 |
| F.L.(A) | 83 | 54 | 51 | 95 | 95 | 96 | 57 | 36 | 29 |
| (B) | 79 | 47 | 22 | 94 | 76 | 42 | 74 | 36 | 8 |

* All concentrations are shown in mg/ml.

Sample B was taken 4 months after Sample A, during which time the patient had continued to receive FAT.

TABLE V.—*The ranking of chemotherapeutic agents according to their* in vitro *activity*

| Maximum | Moderate | Occasional | None |
|---|---|---|---|
| Actinomycin D | Adriamycin | Bleomycin | 6-Mercaptopurine |
| *cis*-Platinum | Cytosine arabinoside | Methotrexate | Procarbazine |
| Cyclophosphamide | Vinblastine | Norethisterone acetate | Warfarin |
| Chlorambucil | | Medroxyprogesterone acetate | |
| Thiotepa | | Methylprednisolone acetate | |
| Treosulfan | | Hexamethylemelamine | |
| Mustine | | Vincristine | |
| 5-Fluorouracil | | | |

lite of cyclophosphamide which was used, showed *in vitro* activity against 50% of the tumours against which it was screened, a figure which approximates to the response rate expected with this drug. The suitability of this compound for screening cyclophosphamide activity *in vitro* cannot unfortunately be proven from the study, since patients did not receive cyclophosphamide. The precursor can be converted either to phosphoramide mustard or to carboxyphosphamide enzymically in the tumour cell and the latter compound shows considerably less cytotoxicity (Connors *et al.*, 1974). Cellular levels of the relevant enzyme are therefore important, and it may be that the use of phosphoramide mustard exaggerates the potential cytotoxicity of cyclophosphamide for a particular tumour.

Specimens were found to exhibit different sensitivities to 6 alkylating agents; complete resistance or sensitivity was rare. These findings parallel those made on the sensitivity of mouse L1210 leukaemic cells to alkylating agents (Schabel *et al.*, 1978) and belie the belief that sensitivity to alkylating agents is an all-or-none phenomenon. In this study it was found that tumours from patients treated with thiotepa showed greater resistance to phosphoramide mustard than did tumours from untreated patients, which parallels the findings that cyclophosphamide-resistant L1210 leukaemia cells showed a 10–100-fold increase in resistance to thiotepa (Schabel *et al.*, 1978). The *in vitro* findings of this study, together with the observation that the toxicity of alkylating agents in combination is not always additive (Schabel *et al.*, 1978) suggests that there may be a role for the combined use of alkylating agents in the treatment of advanced ovarian carcinoma. The apparently greater sensitivity of poorly differentiated tumours to cyclophosphamide may also be of clinical relevance.

Actinomycin D was found to be the most effective antibiotic, as well as one of the most effective agents *in vitro*. The concentration of $5 \times 10^{-10}$ mg/ml which was tested was many times less than the peak plasma concentration achievable in humans of $7 \cdot 5 \times 10^{-5}$ mg/ml (Tattersall *et al.*, 1975) but this was still sufficient to produce >90% inhibition in most specimens tested. The drug would seem to have considerable potential, but in animal models it has been shown that, whilst cytotoxic *in vitro* ($10^{-4}$–$10^{-6}$ mg/ml) it *enhanced* tumour growth *in vivo* (Wilson, 1976). The drug has not been used as a single agent in the treatment of ovarian carcinoma, but has been used with 5-fluorouracil and cyclophosphamide, when it was felt that it was merely adding to toxicity (Barlow & Piver, 1977). The observation that the sensitivity to actinomycin D decreased after treatment with FAT suggests that the agent may be making a useful contribution to cell kill.

The sensitivity of 42% of tumours to hexamethylmelamine was of interest since, although the exact mechanism is unknown, it is thought to require activation *in vivo*, possibly involving N-demethylation by hepatic microsomes (Rutty & Connors, 1977; Rutty *et al.*, 1978). The results suggest either that the drug can exert some cytotoxic effect in its native form, or that some tumour cells are capable of metabolizing the drug to an active form. Procarbazine, another drug which requires *in vivo* activation, never showed *in vitro* activity. Although warfarin has been suggested as having a direct anti-tumour effect (Hilgard & Thornes, 1976) we found no *in vitro* activity against ovarian tumour cells. Progestogens, as has been suggested (Briggs *et al.*, 1967; Malkasian *et al.*, 1977) were found to show some useful activity in a small proportion of tumours, but no *in vivo* data are available on the relevant patients for comparison. Methylprednisolone acetate, again thought to be capable of a direct anti-tumour effect (Liebermann *et al.*, 1977) was found to have some slight activity.

Considerable interest has been centred on the use of the clonogenic assay for *in vitro* screening of the drug sensitivity of human ovarian tumours (Alberts *et al.*, 1980). In the clonogenic assay the chemosensitivity of the "stem-cell" population of the tumour is specifically determined, whereas in a monolayer of tumour cells the chemosensitivity of the total dividing cell population is measured. In advanced ovarian cancer, where rapid debulking is important, it may be that the latter assay is of more relevance, the chemosensitivity of the "stem-cell" population becoming more important in the minimum ($<2$ cm diameter) or microscopic residual-disease situation. Preliminary experiments using cell lines indicate that the clonogenic and monolayer assays can give similar results for a particular drug (Wilson *et al.*, 1981, unpublished data). The monolayer system, with its potential for multiple drug screening, ease of performance and quantitation, may therefore provide a useful tool for the *in vitro* study of the drug sensitivity of human ovarian tumours.

This work was supported by a grant from the Yorkshire Cancer Research Campaign. We are grateful to consultants and theatre staff at Jessop's Hospital for Women, Nether Edge, Northern General, Barnsley District General, Moorgate, Scarsdale and Weston Park for their cooperation in the supply of specimens. Assistance from the Pathology Department, Weston Park Hospital, is also gratefully acknowledged.

### REFERENCES

ALBERTS, D. S., CHANG, S. Y., CHEN, H-S. G. & LARCOM, B. J. (1979*b*) Pharmacokinetics and metabolism of chlorambucil in man: A preliminary report. *Cancet Treat. Rev.*, **6** (Suppl.), 9.

ALBERTS, D. S., CHEN, H-S. G., MAYERSOHN, M., PERRIER, D., MOON, T. E. & GROSS, J. F. (1979*a*) Bleomycin pharmacokinetics in man: 11. Intracavitary administration. *Cancer Chemother. Pharmacol.*, **2**, 127.

ALBERTS, D. S., CHEN, H-S. G., SOEHNLEN, B. & 4 others (1980) *In vitro* clonogenic assay for predicting response of ovarian cancer to chemotherapy. *Lancet*, ii, 340.

BARLOW, J. J. & PIVER, M. S. (1977) Single agent *vs* combination chemotherapy in the treatment of ovarian carcinoma. *Obstet. Gynaecol.*, **49**, 609.

BERRY, R. J., LAING, A. H. & WELLS, J. (1975) Fresh explant cultures of human tumours *in vitro* and the assessment of sensitivity to cytotoxic chemotherapy. *Br. J. Cancer*, **31**, 218.

BISCHOFF, K. B., DEDRICK, R. L., ZAHARKO, D. S. & LONGSTRETH, J. A. (1971) Methotrexate pharmacokinetics. *J. Pharmacol. Sci.*, **60**, 1128.

BRIGGS, M. H., CALDWELL, A. D. S. & PITCHFORD, A. G. (1967) The treatment of cancer by progestogens. *Hosp. Med.* (October), 63.

COFFEY, J. J., WHITE, C. A., LESK, A. B., ROGERS, W. I. & SERPICK, A. A. (1972) Effect of allopurinol on the pharmacokinetics of 6-mercaptopurine (NSC 755) in cancer patients. *Cancer Res.*, **32**, 1283.

CONNORS, T. A., COX, P. J., FARMER, P. B., FOSTER, A. B. & JARMAN, M. (1974) Some studies of the active intermediates formed in the microsomal metabolism of cyclophosphamide and isophosphamide. *Biochem. Pharmacol.*, **23**, 115.

DEDRICK, R. L., FORRESTER, D. D. & HO, D. H. W. (1972) *In vitro–in vivo* correlation of drug metabolism—Deamination of 1-arabinofuranosylcytosine. *Biochem. Pharmacol.*, **21**, 1.

DENDY, P. P., BOZMAN, G. & WHEELER, T. K. (1970) *In vitro* screening test for human malignant tumours before chemotherapy. *Lancet*, ii, 68.

D'INCALCI, M., SESSA, C., BELLONI, C., MORASCA, L. & GARATTINI, S. (1979) Hexamethylmelamine (HMM) and pentamethylmelamine (PMM) levels in plasma and ascites after oral administration to ovarian cancer patients. *Proc. Am. Ass. Cancer Res.* and ASCO, **20**, 185.

FINN, C. & SADEE, W. (1975) Determination of 5-fluorouracil (NSC-19893) plasma levels in rats and man by isotope dilution-mass fragmentography. *Cancer Chemother. Rep.*, **59**, 279.

FRESHNEY, R. I., PAUL, J. & KANE, I. M. (1975) Assay of anti-cancer drugs in tissue culture: Conditions affecting their ability to incorporate $^3$H-leucine after drug treatment. *Br. J. Cancer*, **31**, 89.

HARRIS, P. A. & GROSS, J. F. (1975) Preliminary pharmacokinetic model for Adriamycin (NSC-12312). *Cancer Chemother. Rep.*, **59**, 819.

HILGARD, P. & THORNES, R. D. (1976) Anticoagulants in the treatment of cancer. *Eur. J. Cancer*, **12**, 755.

HOLMES, H. L. & LITTLE, J. M. (1974). Tissue culture microtest for predicting response of human cancer to chemotherapy. *Lancet*, ii, 985.

IOACHIM, H. L., SABBATH, M., ANDERSSON, B. & BARBER, H. R. K. (1974) Tissue cultures of ovarian carcinoma. *Lab. Invest.*, **31**, 381.

LIEBERMAN, A., LEBRUN, Y., GLASS, P. & 4 others (1977) Use of high dose corticosteroids in patients with inoperable brain tumours. *J. Neurol. Neurosurg. Psychiatry*, **40**, 678.

LIMBURG, H. & HECKMANN, C. (1968) Chemotherapy in the treatment of advanced pelvic malignant disease with special reference to ovarian cancer. *J. Obstet. Gynaecol.*, **75**, 1246.

MALKASIAN, J. D., DECKER, D. G., JORGENSEN, E. O. & EDMONSON, J. H. (1977) Medroxyprogesterone acetate for the treatment of metastatic and recurrent ovarian carcinoma. *Cancer Treat. Rep.*, **61**, 913.

OWELLEN, R. J., HARTKE, C. A. & HAINS, F. O. (1977*a*) Pharmacokinetics and metabolism of vinblastine in humans. *Cancer Res.*, **37**, 2597.

OWELLEN, R. J., ROOT, M. A. & HAINS, F. O. (1977*b*) Pharmacokinetics of vindesine and vincristine in humans. *Cancer Res.*, **37**, 2603.

PATTON, T. F., HIMMELSTEIN, K. J., BELT, R., BANNISTER, S. J., STERNSON, L. A. & REPTA, A. J. (1978) Plasma levels and urinary excretion of filterable platinum species following bolus injection and intravenous infusion of *cis*-dichlorodiammine platinum (II) in man. *Cancer Treatment Rep.*, **62**, 1359.

RUTTY, C. J. & CONNORS, T. A. (1977) *In vitro* studies with hexamethylmelamine. *Biochem. Pharmacol.*, **26**, 2385.

RUTTY, C. J., CONNORS, T. A., NGUYEN-HOANG-NAM, DO-CO-THANG & HOELLINGER, H. (1978) *In vivo* studies with hexamethylmelamine. *Eur. J. Cancer*, **14**, 713.

SCHABEL, F. M., TRADER, M. W., LASTER, W. R., WHEELER, G. P. & WITT, M. H. (1978) Patterns of resistance and therapeutic synergism among alkylating agents. *Antibiotic. Chemother.*, **23**, 200.

TATTERSALL, M. H. N., SODERGREN, J. E., SENGUPTA, S. K., TRITES, D. H., MODEST, E. J. & FREI, E., III (1975) Pharmacokinetics of actinomycin D in patients with malignant melanoma. *Clin. Pharmacol. Therap.*, **17**, 701.

WHEELER, T. K., DENDY, P. P. & DAWSON, A. (1974) Assessment of an *in vitro* screening test of cytotoxic agents in the treatment of advanced malignant disease. *Oncology*, **30**, 362.

WHITING, B., MILLER, S. H. K. & CADDY, B. (1978) A procedure for monitoring cyclophosphamide and isophosphamide in biological samples. *Br. J. Clin. Pharmacol.*, **6**, 373.

WILSON, A. P. (1976) *Studies on the drug sensitivities of populations of tumour cells* in vivo *and* in vitro. Ph.D. Thesis, Sheffield.

WRIGHT, J. C., COBB, J. P., GUMPORT, S. L., GOLOMB, F. M. & SOFADI, D. (1957) Investigations of the relation between clinical and tissue culture response to chemotherapeutic agents on human cancer. *N. Engl. J. Med.*, **257**, 1207.

YOUNG, R. C., HUBBARD, S. P. & DEVITA, V. T. (1974) The chemotherapy of ovarian carcinoma. *Cancer Treat. Rev.*, **1**, 99.